# WikiSensing: An Online Collaborative Approach for Sensor Data Management

**DOI:** 10.3390/s121013295

**Published:** 2012-10-01

**Authors:** Dilshan Silva, Moustafa Ghanem, Yike Guo

**Affiliations:** Department of Computer Science, Imperial College London, South Kensington Campus, London SW7 2AZ, UK; E-Mails: d.silva10@imperial.ac.uk (D.S.); m.ghanem@imperial.ac.uk (M.G.)

**Keywords:** sensor data management, online collaboration, collaborative systems, aggregate queries, virtual sensors

## Abstract

This paper presents a new methodology for collaborative sensor data management known as WikiSensing. It is a novel approach that incorporates online collaboration with sensor data management. We introduce the work on this research by describing the motivation and challenges of designing and developing an online collaborative sensor data management system. This is followed by a brief survey on popular sensor data management and online collaborative systems. We then present the architecture for WikiSensing highlighting its main components and features. Several example scenarios are described to present the functionality of the system. We evaluate the approach by investigating the performance of aggregate queries and the scalability of the system.

## 1. Introduction and Motivation

Sensor devices are currently deployed almost everywhere for measurement and surveillance of various attributes of the environment [1]. A sensor can be defined as a device capable of capturing physical information such as heat, light or motion about a physical system or an environment. These sensor devices can provide readings of many properties such as pollution levels, temperature and road traffic. This research focuses on sensors that produce data streams that consist of a sequence of values (sensor readings) and timestamps. A sensor network is a collection of sensor nodes that collectively measure environmental changes. Sensor nodes take measurements and store them on-board or relay data towards remote systems [2]. With the growth of sensor networks, new technologies are required to systematically manage the sensors data. Stream data is large, real-time and continuous [3].

The use of online collaboration has largely proven to be an extremely powerful principle for sharing and gathering information with many advantages as explained by [4]. The objective of this research is to incorporate collaboration into sensor data management system to help reduce the effort of the users and help in exploiting their knowledge and experience in the management of sensor information.

### 1.1. The Challenges

The challenges of designing and developing a collaborative sensor data management system can be categorized as data management challenges and collaborative challenges.

#### 1.1.1. Data Management Challenges

The data management challenges are directly related to managing large amounts of sensor data. Providing efficient and scalable storage is vital for a sensor data management system. The ability to organize the stream data for querying these large volumes of sensor data and scaling the system to handle a significant amount of sensor devices without compromising performance are also important aspects. The data management challenges can be categorized as follows:

- **Infrastructure** Designing a framework for scalable, efficient storage and retrieval of sensor information. The framework must be capable of efficiently storing and retrieving large volumes of sensor information. It must have the capacity to scale in order to handle large number of connected sensors that periodically submit data as well as a large number of users that concurrently access the system.
- **Querying** The need to deal with both real-time and historical information. Querying constructs are required as this information arrives to the system continuously. The real-time nature and the continuous flow of sensor data have created the requirement for a near real-time processing of such data. The challenge arises when a query is processed and an output is produced, more up-to-date data arrive making the previous reading out-of-date. For instance assume that a query completes processing using a window of real-time data at the time frame *t1*. This output will be invalid at time frame *t2* (where *t2* > *t1*) as new data would have arrived. Also query constructs are required to mine historical information, when, for example, a user may wish to investigate sensor readings from a previous time.
- **Information** The need for aggregating data from multiple sensors as well as data from reference data sources. The different reference sources being sensor data providers such as the meteorological (e.g., www.metoffice.gov.uk) or transport (e.g., www.tlf.gov.uk) departments. Aggregation is required to combine data streams with each other to obtain combined readings and to combine data streams with reference data to obtain aggregated information. The challenge in aggregating different data streams arises due to the disparity of the sensor types, measurements, accuracy, quality of readings and time frames. For example consider the combination of two temperature data streams that have different unit of measurements (Celsius and Fahrenheit) and are submitted in different frequencies and hence have different time points.

#### 1.1.2. Challenges in Collaborative Environments

The collaborative challenges are related to organizing the collaborative data and accounting the creditability of this information by online users. The collaborative challenges are described as follows:

- **Organization of information** The challenge of organizing the sensor data and the information provided by collaborating users. The sensor data primarily contains the data streams and the sensor meta-information. Knowledge and experience of the collaborators would be about the information of the sensor devices (characteristics of sensors) and information on their deployed locations. It is a challenge to organize sensor information as different users have diverse goals, views and therefore provide different annotations and this cannot be accommodated in a fixed schema. Further the need to link different types of information is required when organizing information when collaborating online.
- **Assess trustworthiness of information** The need to assess the trustworthiness of the sensor data streams as well as the user annotations. For instance identifying whether the sensor information is correct or accounting the credibility of the annotations and comments provided by the users. The difficulty of assessing the trustworthiness of a data stream or an annotation is due to the openness of online systems that permits any user to add and edit information.
- **Manage conflicts** An important collaborative challenge regarding the trustworthiness of the information is managing conflicts between contradicting information. For example two sensors that are deployed at the same location may have substantial differences in sensor readings or there may be conflicts between annotations from two separate users. Dealing with conflicts are challenging due to the lack of information that can be used to support the conflicting sources.

### 1.2. Approach and Contributions

We summarize our main approach and contributions towards addressing the above challenges as follows:

- A model for a collaborative sensor data management system. This model provides interfaces for online collaborators, a middleware containing the business logic needed for the sensor data management and a storage model suitable for the efficient storage and retrieval of large volumes of data. This architecture also contains the modules needed to maintain a media wiki for the management of collaborative information.
- A working prototype system with a web interface, Wiki pages and a service layer for users to connect sensor devices and to add, retrieve, annotate and share sensor information. The system is developed using a hybrid storage strategy to support vast amounts of sensor readings. The implemented application layer of the system provides the business rules to manage and control access to the underlying data. We also introduce a set of query constructs providing the special operations that are required for obtaining the sensor information.
- Case studies to demonstrate the functionality and usage of the system. These case studies describe the aspects of organizing information, registering sensors, creating virtual sensors and assessing the trustworthiness of data in WikiSensing.
- An experimental evaluation of the strategies used by WikiSensing to aggregate data streams to create virtual sensors. This evaluation is geared towards illustrating the effectiveness of the approach adopted in the system.

### 1.3. Outline of Paper

The remainder of this paper is organized as follows: Section 2 describes past and current work that is relevant to this research. Section 3 presents the system architecture, storage model, query constructs and API web services of WikiSensing. Section 4 explains the implementation of the basic functionality, the creation of virtual sensors and the collaborative challenges of trustworthiness and conflict management. Section 5 presents several case studies of WikiSensing. An evaluation of the system is explained under Section 6. Sections 7 and 8 contain a discussion, avenues for future work followed by the conclusion summarizing the research work.

## 2. Background and Related Work

### 2.1. Sensor Data Management Systems

It is quite natural that sensors produce a vast amount of data as they continuously monitor environments [3]. The extremely high volume of sensor data poses a challenge for data management systems. We explain three popular sensor data management systems namely the Aurora system, the COUGAR system and Cosm. While Aurora and Cougar are predominantly research-based systems, Cosm is currently available online system with API support.

Aurora is a Database management system for managing data in monitoring applications [5] developed by the Universities of Brandeis, Brown and MIT. The Aurora system processes incoming data streams by passing them through a data-flow system which then outputs a stream that can be used by applications. The Aurora query algebra contains several primitive operations for expressing queries. Queries can be executed while the input tuples are run through this data-flow system. For instance, the filter operator that applies any number of predicates to each incoming stream and the aggregate operator that applies a function across a window of values in a stream. Once an input has worked its way through the paths of the flow it is generally drained from the system. Aurora can also maintain historical storage in order to support certain *ad-hoc* queries based on a persistence specification.

Developed by Cornell University the COUGAR system [6] is a sensor data management system that supports querying of the sensor data. It follows a distributed query processing approach where the query workload determines the data that should be extracted from the sensors. The COUGAR system uses an object-oriented database for storage and it models each sensor as a new Abstract Data Type (ADT). The stream processing functionalities are designed as ADT functions that return sensor data. The Cougar system also supports long running queries formulated in SQL by extending the query execution engine with the new construct *every* (<Time frame>).

Cosm (www.cosm.com), formally known as Pachube, provides a platform to connect devices and products with applications to provide real-time control and the management of sensor data. The online web-based system enables users to register sensors and attach data streams to it. It provides an API to manage sensors data on its remote platform. According to their web site it manages millions of data points from thousands of individuals, organizations and companies around the world. The sensor information that Cosm stores is based on sensor environments, data streams and data points.

### 2.2. Collaborative Sensors

The work in [7] presents a system with collaborating sensors using a sensor grid framework and a sensor grid client which is a collaborative session that enable meeting participants to share sensor information. These multiple collaborative sessions can interact with any combination of deployed sensors via this sensor grid. Collaborative sensor grids are a combination of sensor networks and grid computing. In this model each sensor gathers information from the environment and publishes it in real-time. A sensor adapter retrieves data from a connected sensor and communicates it to the sensor grid. The adapter provides among other capabilities a service interface to each sensor which facilitates the Grid integration and the Web service based management framework. This sensor adapter processes the raw sensor data and outputs the refined information.

The QuakeSim web service environment [8] integrates real-time and archival sensor data with high-performance computing applications for data mining earthquake data. This distributed computing infrastructure consists of Web services that provide access to data through well-defined programming interfaces (expressed in WSDL (www.w3.org/TR/wsdl)).

The research work by [9] describes virtual sensor networks based on collaborative wireless sensor networks. They define a collaborative virtual sensor network as a subset of sensors that collaborate to carry out a given application. These virtual sensor networks may exist simultaneously on a physical wireless sensor network, and the membership of the sensors may change over time. An area of this work is geographically overlapped applications. For example consider a set of sensors that are deployed to monitor rock slides and animal crossing within a mountainous terrain. The motivating factor is to have resource sharing where different types of devices that detect these phenomena rely on each other for data transfer without having to deploy separate networks. Similarly a goal of this research is to use the readings of existing sensors to obtain information where sensors are not currently deployed without the need of physically deploying them.

### 2.3. Wiki Approaches and Rating Methodologies in Online Collaborative Systems

A wiki is a system whose users can add, modify, or delete its content via a web browser using a simplified markup language [10]. This approach has enabled quick access to information [11] and the rapid production of data. Systems such as Wikipedia (en.wikipedia.org) and WikiPathways (www.wikipathways.org) are examples that successfully implemented the Wiki approach. So ideally what we stride for is a sensor data management platform that gathers sensor readings, uses collaborators to annotate information and follows a Wiki method to enable this online collaboration.

StackOverflow (Stackoverflow.com) and BioStar (biostar.stackexchange.com) are online comment based systems specialized in answering specific questions. While StackOverflow focuses on computer programming-related problems, BioStar mainly concentrates on biology-based issues. These systems enable users to post their questions online where experts are able to provide feedback by adding comments.

These systems have proved to be a popular method of getting domain specialists around the world to comment and provide solutions to specific problems. As this is only a comment based system the user with the actual question has to manually distinguish the comments and use their own judgment in order to come to a certain conclusion. There are several differences between these comment-based systems and a question answer sites such as Yahoo Answers. The information on the question as well as the posted comments that may amount to the answer can be edited in StackOverFlow or BioStar. This enhances the collaborative power in dealing with specific problems as it ensures that the information in the questions and comments are more up to date.

These systems use the concept of tags which are keywords or labels that categorize a question with other, similar questions. This makes it easier for others to find and answer your question. It keeps track of the unanswered questions in the system and ranks them in accordance to the number of users who viewed them. This drives the attention of users to answer these questions as the popularity and importance are highlighted.

StackOverflow and BioStar, uses a rating system to assess the reputation of a user. This is based on the number of questions answered, edited posts and the scores rewarded.

### 2.4. Online Collaborative Systems

Online collaborative systems in the nature of the Polymath Project (http://polymathprojects.org/) and the OpenStreetMap (www.openstreetmap.org) provide powerful infrastructures allowing people obtain and share information. They have become the basis of knowledge sharing among users.

The OpenStreetMap, a freely available map that covers the whole world allows users to view, edit and manipulate geographical data in a collaborative manner [12]. It uses the knowledge on location information such as road, pathways and buildings provided by the users to build up comprehensive geographical maps. With over 320,000 contributors OpenStreetMap is a geographical source that provides data on maps without any technical restrictions on their use. It acquires data when the contributors provide location information using devices such as GPS, cameras and own observations. Similar to Wikipedia, OpenStreetMap enables any interested user to provide information.

OpenStreetMap has a set of rules for sharing knowledge that are based on simple logic which have proved to become extremely effective in the online collaboration process. For example data provided on a route within a short time period of a GPS signal is considered less accurate hence data received on routes taken from bicycle is deemed to have prominence over data received from a relatively faster moving car.

OpenStreetMap obtains the knowledge from its users to annotate its maps, where the goal is to get the input of the users who are most familiar with these routes. Similarly in sensor data management a user who has knowledge about the local area would be more suitable to provide information on certain factors that would affect the reading of a particular sensor. For example, imagine a temperature sensor that is located in a building. A person who works or lives at that location would best know if there are certain factors affecting its reading such as a refrigerator or a heater. The knowledge of locals is a vital aspect as sensor devices can be located around the globe and there may be several factors influencing their measurements.

The Polymath project is an online comment based systems created to test if mass collaboration can be used to solve mathematical problems [13]. The method used to support the Polymath project was to use the commenting system in a blog and devise a series of rules [14] to govern how contributions should be made. The project was successful with over 40 people having contributed and resulted in at least two new publications. This concept continues to develop and has created over seven new Polymath projects to resolve various mathematical problems. Moderating and measuring of contributions, safeguarding the participant's reputation and continual building of social connections that were based on the behavior and psychology of participants are considered as the key aspects that lead to the success of this project. Users can contribute to the project by providing comments based on their knowledge and experience. The users collaborate with each other by these comments that create a discussion where ideas are instituted, exchanged and criticized.

### 2.5. Conflict Management in Online Collaborative Systems

Conflicts are a common problem in the case of collaboration and managing these conflicts is an important challenge when designing collaborative systems [15]. Conflicts occur when two or more sources provide contradicting information on a specific domain. Examples of conflicts can be in the nature of disagreements on attributes such as the total worldwide box office gross of a movie or the height or depth of a specific location or disagreements on a name, location, or the actual spelling of a term.

There are several steps involved in managing conflicts. The first step would be to detect a conflict. The underlying conflict may have different levels of complexity. A simple conflict for example could be in the nature of a disagreement based on a single name or a value. Secondly managing conflicts involves evaluating the conflicting situation. To evaluate a conflict a set of policies are required. These policies can be based on attributes such as the reputation and credentials of the collaborating users. Consequently a weighting can be given to the conflicting views. Based on this weighting a decision can be made on which candidate or opinion should be supported.

A conflict in the context of Wikipedia is when information on a page is modified using contradicting information by another user, for example, conflicting statements on articles. Wikipedia has a dispute resolution mechanism [16] in order to resolve conflicts between editors. This is a manual process that provides a set of guidelines that must be followed in the case of a conflict or dispute. Dispute resolution requests lead to discussions with editors in a discussion page relating to that particular article. It is then the responsibility of the administrators with access to restricted technical features such as deleting and restoring pages to be neutral in resolving the disputes based on the actual contents of the discussion.

## 3. The Overview of WikiSensing

This section describes the layered architecture of WikiSensing highlighting the main components and their interactions. We also present the WikiSensing hybrid storage model and the supported query constructs.

### 3.1. Overall Architecture

**Figure 1.**
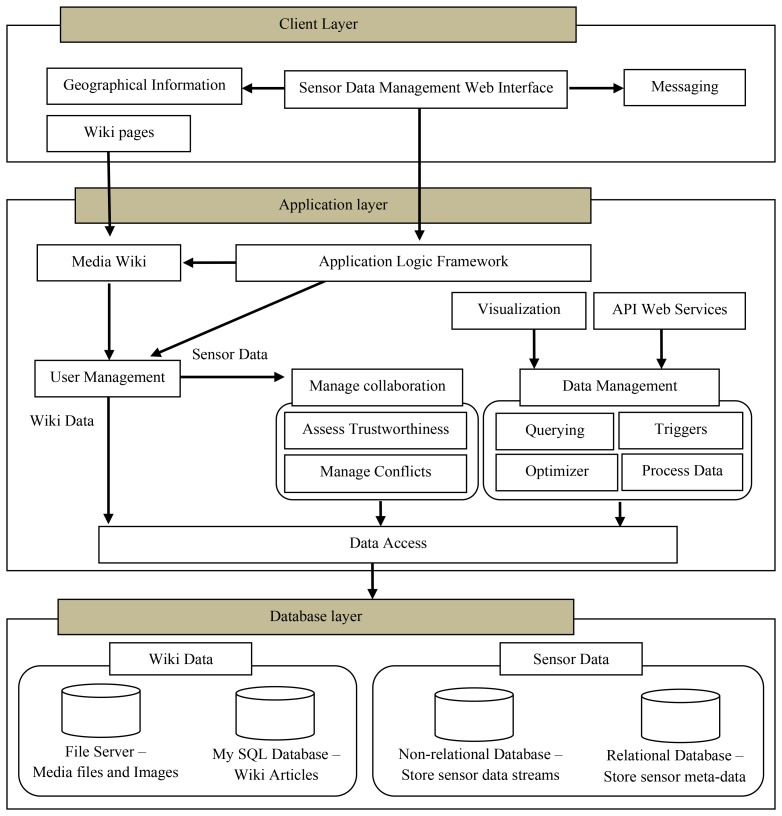
The architecture of WikiSensing.

Figure 1 illustrates the architecture of WikiSensing including the main components that provide its functionalities. The WikiSensing system is designed based on a three-tiered architecture consisting of a database, application and client layer.

The database tier hosts the databases for the sensor and the wiki data. The application layer directly interacts with the database layer via the data access module. The application tier contains the business logic for the API services, controlling user access, managing the data and supporting online collaborations. The client layer interfaces the user by providing a web interface for sensor data management and a Wiki front to enforce the sharing of knowledge and information.

#### 3.1.1. Database Layer

The wiki data for the sensor information are stored in a MySQL database and any media files including images and videos are stored separately in a file server for efficient access. The textual information of the wiki pages can be stored in multiple languages in a database server.

The sensor data is stored using a hybrid database strategy where the sensor meta-information is saved in a relational database the comparatively large amount of sensor reading reside in a non-relational database.

#### 3.1.2. Application Layer

The application layer or middleware of WikiSensing comprises several components that are collectively responsible for the control and the management of the data and the user. These components predominantly contain the rules and the algorithms that are required for the functioning of the system.

The application logic framework is built on an ASP .NET framework. The WikiSensing sensor data management business logic is also based on this ASP .Net framework that implements the model-view-controller [17] software design. The main advantage of using such a framework is based on its clean separation of functionality [18] that is required for implementing WikiSensing's layered architecture. The ASP .NET framework supports user management for the web application. The functionality includes validating user credentials, creating and modifying membership users, and managing user settings such as passwords and e-mail addresses. The application logic contains the operations that coordinate and invoke the functionalities of the other modules within the application layer. For instance it executes the operations that register a sensor device in the data management component and automatically creates a corresponding Wiki page using the media wiki.

The Media Wiki component (www.mediawiki.org/wiki/MediaWiki) that hosts the Wiki runs on a PHP (www.php.net) framework on the application server. The PHP framework implements the security policies and rules that are prescribed by the media wiki for the user management of the wiki users in order to control access to its information.

The manage collaboration component controls the collaborative data by obtaining the sensor meta-information, sensor readings and background details of the users. This information is used to assess the trustworthiness and to manage conflicts of the user annotation and the readings of sensors data streams.

The data management module supports querying, setting up triggers on data streams and validating and verifying the data that is stored in the system. This further contains logic to optimize querying in order to enhance the performance of aggregation of virtual sensors. The main forms of querying can be categorized into regular queries that select sensor details such as sensor readings and its deployment information and aggregation queries that combine several data streams. The system also supports continuous queries that provide readings to the users uninterruptedly within a specified time period. Triggers are mainly used to inform users when a certain threshold has been reached on a particular data stream. This is useful to provide alert in the case of abnormal or unusual behaviors of sensor readings. The system contains the functionality to process the data that is submitted and returned from the system. These business logic rules validate the inputs of the user and to process the data that presented by the system. For example the logic to create the output readings for virtual sensors reside in this component.

The optimizer module focuses on increasing the efficiency of the aggregate queries which are considered as one of the most common operations in sensor data management [19]. It is responsible for analyzing the information that contains the data streams that constitute the virtual sensors and identifying the most efficient (with minimal amount of database reads) methodology for aggregation. This also controls the storage of the virtual sensor readings in a cache repository for quick access.

The Visualization module functions to provide graphical representations of the sensor data streams, primarily using charts. The API web services exposes the functionalities of WikiSensing in order to be used from different programming platforms. The web service provides functionality for users to connect to the WikiSensing middleware using different technological platforms. These services access the data from the underlining database server via the business logic imposed by the data management module. The Data Access component contains the operations for reading and writing the data to the database layer. The data management and collaboration components access the databases via the Data Access module.

#### 3.1.3. Client Layer

The front end of the system is a graphical user interface consisting of a series of web pages and Wiki pages. The main web interface is implemented using ASP .NET 3.5 technologies. The Wiki pages are created using a media wiki that runs in the in application layer.

The geographic information component is an external system such as Google maps API (code.google.com/apis/maps/index.html) that enables the users to incorporate location information. The messaging component is implemented using PostBin (www.postbin.org) that facilitates users to register certain URLs so that asynchronous requests can be logged when events occur. The PostBin is exclusively used in WikiSensing for sending messages in the case of triggers.

### 3.2. The Data Model

**Figure 2.**
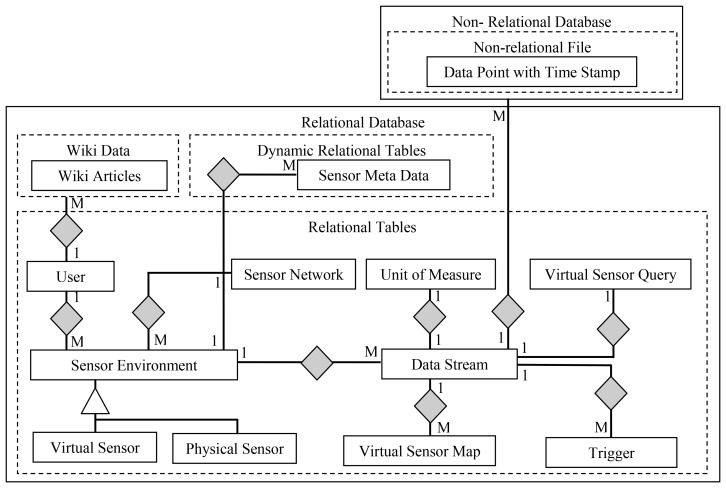
The WikiSensing Hybrid Data Storage Model.

Figure 2 depicts entity relationship diagram for the WikiSensing hybrid storage model. The WikiSensing data storage comprises of a relational MYSQL database to store the environment details, Sensor meta-data, virtual sensor details and user information and a relation free MongoDB [20,21] to store the data points and time stamps of the data streams.

The main motivation behind selecting this hybrid approach is twofold. Firstly using a high speed database to store the vast number of sensor readings would enhance the performance. MongoDB is a document-oriented, schema free storage system that uses memory-mapped files [22]. It is a relational free database that provides better performance to a relational database such as MYSQL [23]. Secondly as non-relational databases such as MongoDB lack the atomicity, consistency and durability properties [24] it is not suitable to store information that requires a degree of concurrency control. The primary aim of MongoDB is to be lightweight and fast and does not use traditional locking or complex transactions with rollback [25]. While WikiSensing encourages online collaboration certain data (for example, environment information, and sensor meta-data) are frequently updated as they are exposed to several users. Hence atomicity is required so that if a part of the transaction fails, the entire transaction is aborted, and the database is left in a consistent state. The consistency property ensures that any transaction brings the database from one valid state to another while durability is the guarantee that all updates are reflected (physically written) to the database. Hence WikiSensing uses the MYSQL database that guarantees these important transactional properties to store the meta-information. The system uses MongoDB to store the sensor readings as Key Value Pairs (A unique Key and a Value which is the sensor reading and timestamp) which is not be updated but only be inserted and would not require such transactional properties.

#### 3.2.1. Relational Tables

The Sensor Environment, Sensor Network, Data Stream, Trigger, Virtual Sensor Map, Virtual Sensor Query, Unit Of Measure, User are relational tables, and the Sensor meta-data is a dynamic relational table that resides in MYSQL.

The Environment table which maps on to a deployed sensor contains the geographic detail of a location that the sensor devices are deployed in. Sensor environment are specialized into physical sensors and virtual sensors. Virtual sensors contain specific information on whether the storage of sensor reading is persistent or calculated dynamically. The information of the contributing sensors of a virtual sensor is maintained in the Virtual Sensor Map table. This strictly contains an identity of the virtual sensor and the list of identities of the sensors that contribute towards it.

The Data Stream maps to an actual sensor that contains the type and reading information of that device. An environment can have multiple data streams. A single data stream can have multiple triggers imposed on it and this information is stored in the trigger table. The sensor Network contains the details to group a set of sensors that belong to a specific sensor network.

The user table stored the credential of all active users of the system. This generally contains the qualification, experience and the contributions by each user. This entity links the wiki data relation of the Media wiki that contains the wiki information. The table Unit of Measure contains the predefined measurement units as well as the units defined by the users. This also stores a conversion function to a specified base measurement unit.

#### 3.2.2. Dynamic Relational Tables

The primary source of the sensor meta-information is stored in the Sensor Meta Data table. This contains extensible information due to different sensors having different characteristics that define various properties and capabilities of a sensor. This information is categorized into three main fields the sensor attributes, sensor specifications and user defined values. Data in these columns are stored as an attribute name, attribute value pair. Users are free to add their own attributes and this information are stored as user defined values. Each sensor data stream of a particular environment has a sensor meta-data record. This table is defined as dynamic table with variable-length columns which saves disk space due to the extensible nature of the sensor meta-information. The following example illustrates the attribute string structure stored in the variable sized columns:

<*Accuracy*>:<*value*>;<*Precision*>:<*Value*>;<*Sensitivity*>:<*Value*>;<*Resolution*>:<*Value*>

#### 3.2.3. Non-Relational Data

The non-relational data is stored in a relational free MongoDB. MongoDB stores this information as a collection which is analogous to tables in a relational database. This is the Data Point information that contains the sensor readings and corresponding time stamps. The Data Point also contains a uniquely generated key and includes the environment identity and the data stream identity to link it with the entities in the relational database.

### 3.3. WikiSensing Query Constructs

The WikiSensing query language selects data from a combination of relational (MySQL) and non-relational (MongoDB) data. The constructs that are introduced are prefixed with the term *wiki* for distinction. The query language is SQL like and is implemented in the Data management module of the application layer. The following illustrates a sample structure of a query that is supported by WikiSensing. The following SQL example cites the constructs that are newly introduced:
*SELECT* <*List of Attributes*>*FROM* <*List of Relations*>*WHERE* <*Condition*> *WIKI_LOCATION* <*Coordinates OR Location name*> *WIKI_RADIUS* <*Specified in meters*> *WIKI_WINDOW* <*Window specified by time OR Number of readings*> *WIKI_UOM* <*Converts to*> *WIKI_PROPORTION ON* < *[Distance], [Time]*> *WIKI_SAMPLE_STREAM* *WIKI_CONTINUE_FOR* <*Time specified in Hours*>

The construct *WIKI_WINDOW* indicates a time window for the sensor readings specified in hours or minutes which selects the readings within a specified time period prior to the execution time or a record size window that selects the most up to data sensor readings from the data stream specified by a number. *WIKI_PROPORTION* construct is used to indicate that the aggregated values must be based on the weighted mean of the specified attributes. The system currently supports linear aggregations such as averaging and summing of data streams. The time frame or the distance or both can be specified with this construct to obtain a weighted mean. The *WIKI_LOCATION* construct select records with in a location or more specifically with in the specified coordinates. *WIKI_RADIUS* can be used in conjunction with the *WIKI_LOCATION* construct to specify a radius so that it selects records within a particular radius (in meters) to the specified location or coordinates. The *WIKI_SAMPLE_STREAM* construct samples the data streams to match the stream with the largest frequency when aggregating multiple data streams. *WIKI_UOM* is also used in aggregation queries specifies the base unit of measure. The construct *WIKI_CONTINUE_FOR* returns values from the query continuously for the specified time period.

## 4. The Features of WikiSensing

In this section we describe some of the main features of WikiSensing by referencing the architecture that was explained in the previous section. We primarily focus on the functionality of the virtual sensors, API web services, collaboration and trust and conflict management.

### 4.1. Virtual Sensors

A virtual sensor is a sensor that is not physically deployed at a certain location but uses data streams of nearby located sensors to obtain sensor reading. Virtual sensors can be implemented by selecting a set of contributing sensor data streams, either by using the web interface or the application services. These aggregations are linear operations that produce a single value or a data stream. This is an extremely useful feature that provides sensor readings in the case where no physical sensors are present at a specific location and even where combining a set of high quality sensors can lead to a higher accuracy reading.

The existing sensor data is used to create this conceptual item of a “virtual sensor”. This would require the knowledge and experience of the collaborating users for example, the knowledge of geographical locations, or the reliability factors of the sensor devices. The knowledge of the collaborating sources are used to annotate sensors so that they can be combined to create aggregated virtual sensor in a logical manner. Virtual sensors are most useful in cases where a user requests a sensor reading (Temperature or pollution level) where a physical sensor is not deployed as well as in situations where a low quality sensor is physically deployed, but the aggregation of a set of high quality sensors that are located nearby may lead to a more accurate reading.

**Figure 3.**
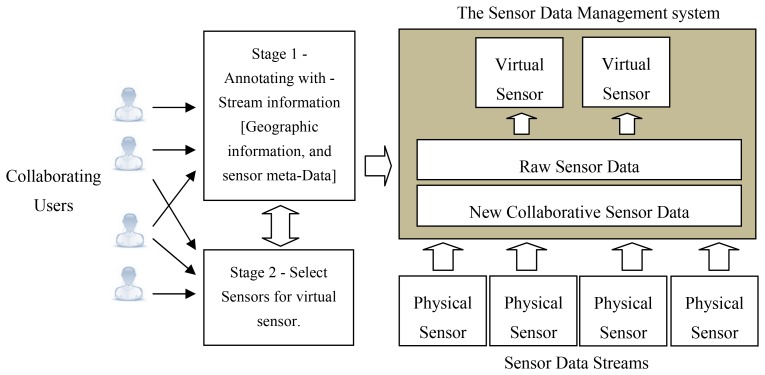
Collaborating sensors to create virtual sensors.

Figure 3 illustrates a scenario where several physical sensor devices are combined to create a virtual sensor. During stage 1 of this process the collaborating users are involved in annotating the sensor streams with geographical information and sensor meta-data such as reliability, precision and accuracy. This information is recorded in the wiki. Stage 2 involves the users selecting the physical sensors that would contribute to the virtual sensor. The readings of the virtual sensors can either be persistent or calculated dynamically. The query involved in aggregating the data streams for the virtual sensor can be updated to increase or reduce the scope of the sensor or modify the valid window size.

The components that deal with the creation of the virtual sensors are contained in the data management module. The functionality spans from querying for deployed sensors, registering a virtual sensor, to selecting the contributing sensors. The API web services component is used to connect the deployed sensors to acquire the sensor reading into the system. These readings are processed and stored in the database via the data access component.

### 4.2. API Web Services

WikiSensing supports a list of API web services that can be used by external platforms to automatically connect sensor devices to the system. These services can be categorized as inputs such as submitting data, or as queries that produces an output. The services to create environments, data streams, triggers and add data points to existing streams submits data to the system. The querying services include listing user environment, outputting sensor streams in formats such as XML or JSON and obtaining minimum, maximum and current values of a data stream.

To access the web services the user is required to obtain a reference to the API. The following example code snippet written in C# illustrates obtaining a WikiSensing service reference.

*WikiSensingServiceReference.WikiSensingAPISoapClient ClientWebreference* = *new TestWebService.WikiSensingServiceReference.WikiSensingAPISoapClient()*;

The API web services are written into the web services component which resides in the application serves and communicates with the database layer through first the data management and then the data access component.

### 4.3. Collaboration

The success of a collaborative system is centered on the usability and the organization of the information. In order for users to collaborate, the system must contain the required structure to enable sharing of knowledge and information. The aim is to use a Wiki infrastructure and the challenge is to make it successful for collaboration of sensor information.

**Figure 4.**
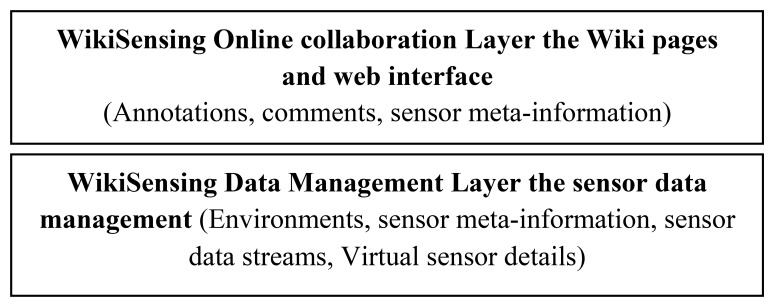
The WikiSensing Information Layers.

WikiSensing has enabled collaboration through the use of a combination of Wiki and web pages that enables users to add annotations, comments and sensor meta-data to the sensor information. The collaboration layer sits on top of the Data management layer as depicted in Figure 4. For example, the users can annotate and comment on the sensor environments, sensor meta-data and the data streams that are managed in the sensor data management layer.

There are two interfaces that an online collaborator can use to provide information. The first interface is the Wiki pages that are automatically created for each sensor and its deployed environment where the users can provide annotations and comments on this content. Secondly the WikiSensing web pages that directly correspond to the information stored in the underlying database. These web pages, for example, are used to register new sensors, create virtual sensors, add or update sensor meta-information.

Online collaboration is enabled in the form of Wiki pages on the client that are hosted using a media wiki deployed in the application layer. These wiki pages contain the information that the uses add or update. All updates are logged and access to the Wiki articles is controlled by the user management component.

### 4.4. Trust and Conflict Management

Trust management in WikiSensing is based on a rating scheme that is calculated using the following information:

- The meta-data of the sensors to calculate the s*ensor reliability rating*.
- Aggregated reading of nearby sensors to calculate the *distance of the sensor readings*.
- User credentials and contributions to calculate the *user reliability rating*.

The information to calculate the sensor and user reliability rating is taken from the sensor meta-data and user information. For example, a sensor with properties such as a good accuracy and precision would have a higher reliability rating than a weaker sensor. A standard method is initially set to define the sensor properties as illustrated in the Wiki page for sensor meta-data in Figure 9. This page contains a list of attributes such as accuracy, sensitivity that can be used as metrics to calculate this rating. Hence a set of general rules are used to evaluate this information in order to obtain a standardized rating that is used throughout the system. The ratings are in the scale of 1 to 10. It is common practice to update (recalculate) these ratings with the addition of new information. Once requested by the users these ratings are reordered in the corresponding Wikipages. For example, the sensor reliability rating is listed in the wiki page that contains the meta-data of a sensor.

The distance of the sensor readings is calculated using the data streams stored in the system. For instance, if the users want to check the trustworthiness of a particular data stream, they can do this by aggregating several nearby data streams and obtaining the difference between the aggregated reading and the actual sensor reading. The difference of the readings and the list of selected sensors are recorded in the Wiki page of the relevant sensor.

**Figure 5.**
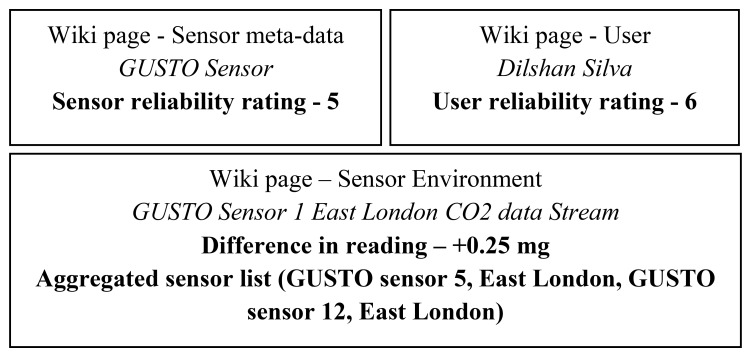
Example of the sensor, user reliability rating and difference of the sensor reading.

These ratings and values (Figure 5) can be compared by the users to assess the trustworthiness of the information. The ratings can then be further used to manage conflict that exist between data streams or conflict between user annotations.

The Manage collaboration component includes the sub modules that contain the operations to assess trustworthiness and manage conflicts. For example, the sensor reliability rating is calculated by obtaining the sensor meta-data. Once the information is acquired and the reliability rating is calculated, it is stored with the sensor data. This information is then automatically recorded into the Media Wiki by the application logic framework.

## 5. Case Studies

In this section we present four different case studies that illustrate the use and the functionality of the system. The first case study describes how information is organized in WikiSensing. The second shows how multiple sensor data streams are aggregated. The third scenario is focused on creating virtual sensors using the system, and the last case study demonstrates the manner in which trustworthiness is assessed in WikiSensing.

### 5.1. Case Study 1: Organizing Sensor Information

Stage 1: Registering an Environment for a sensor in the system
  - The first mandatory step for registering sensors is to create an environment that the sensor is deployed in. This information (Table 1) includes location descriptions, for example, name of the city, street and country as well as geographical coordinates which are the longitude and latitude that can be selected using the provided Google map.
  - The user is also encouraged to provide a feed description that contains the type of sensor, for instance, type GUSTO [26] sensors. User can create private feeds, which is only be visible to them as well as public feeds, which are accessible by the other users of WikiSensing.

**Figure 6.**
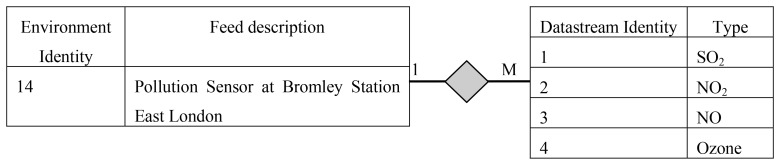
The multiplicity between the environment and its data streams.

**Figure 7.**
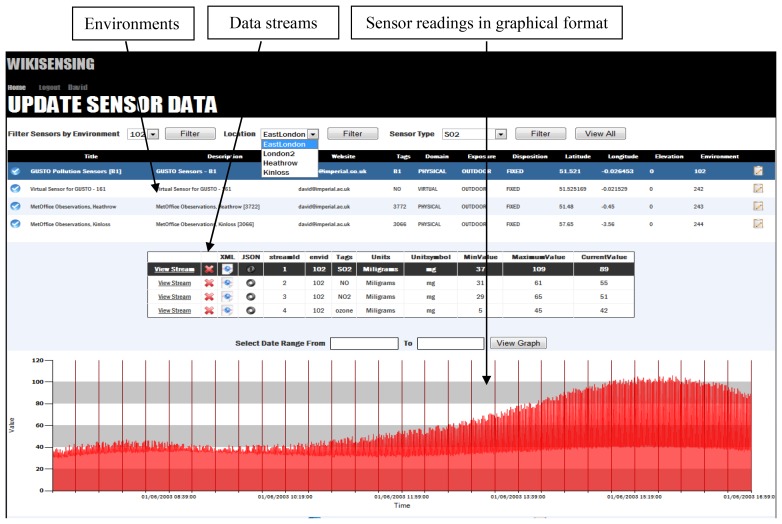
WikiSensing graphical view of sensor data streams.

**Figure 8.**
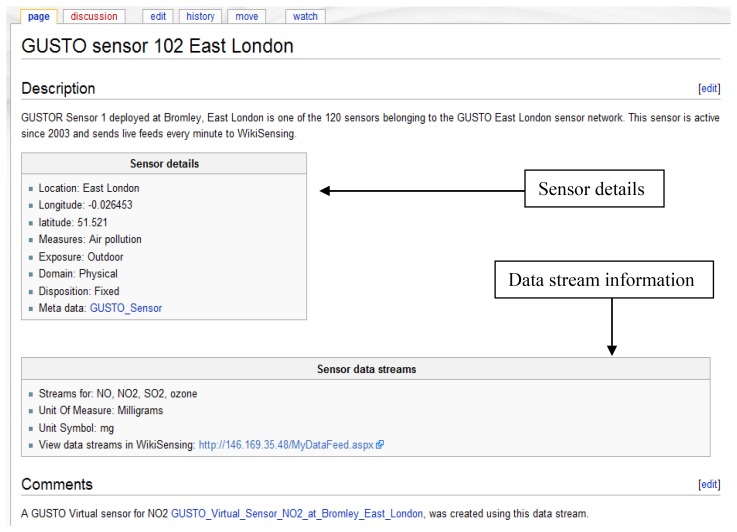
Wiki pages that record the sensor and data stream information.

**Figure 9.**
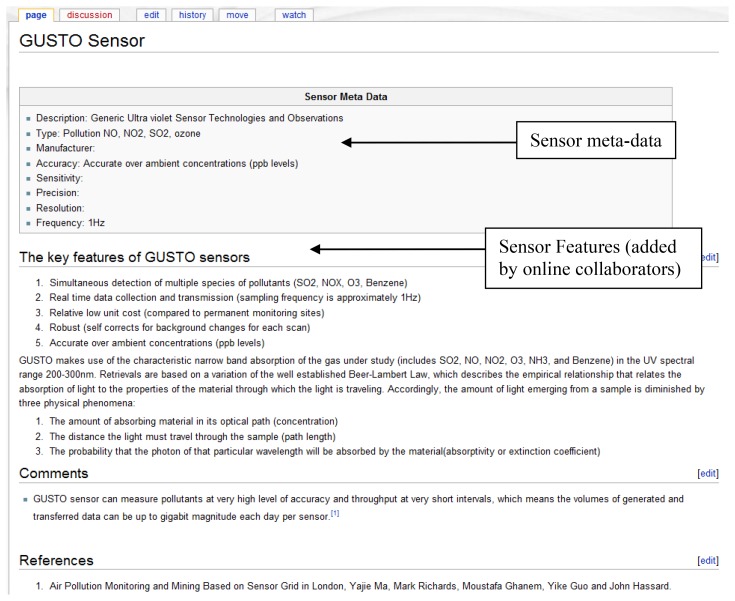
Wiki pages to record the sensor meta-data.

Stage 2: Registering the data streams of a sensor
  - A sensor in an environment can measure several attributes and produce multiple data streams (Figure 6), for example, a sensor with data streams that measures NO, NO_2_, SO_2_ and ozone (GUSTO sensors). The data streams are representations of a physical or virtual sensor that is deployed at a particular location.
  - Once an environment (deployed sensor) has been defined and a data stream (a sensor can have multiple data streams) is attached to it, data points can be added. The data stream information can be viewed graphically as illustrated by Figure 7.
  - The measurement units for a data stream can either be selected form a predefined list or can be explicitly specified by a user. When defining a new unit of measurement the user is required to provide a conversion function to a base unit.
  - A wiki page is created automatically and the provided information is recorded (Figure 8). This page contains a description of the sensor environment followed by the sensor details and information of the sensor data streams. The system also automatically links the environment with a page that contains the relevant sensor meta-information (Figure 9). This Wiki page lists the sensor meta-data and the features of the sensor that can be updated by collaborating users. The user is able to create a new sensor meta-data Wiki page in the case where a corresponding page does not exist. These Wiki pages are automatically updated when the corresponding information on the system are modified by the user.
  - Once the location information is provided the user can then connect the sensor data streams to the system via the web service layer. This is done by obtaining a web service reference of the WikiSensing web service through any programming platform.
  - The Wiki page displayed in Figure 9 shows the inclusion of referencing the information added to the page by the user. In this example the user annotates a GUSTO (Generic Ultraviolet Sensor Technologies and Observations) sensor by referencing a research paper [26].

Stage 3: Query sensor the data streams
  - The following is a sample query that averages the readings of a single sensor for a window size of 1 hour. The construct *WIKI_WINDOW* indicates a time window for the sensor readings specified in hours which selects the readings within an hour prior to the execution time.
*SELECT Average (Value)**FROM Environment, Datastream, DataPoint* *WHERE environmentId* = *‘14’* *AND streamType* = *‘NO2’* *WIKI_WINDOW 1h*

Stage 4: Registering a sensor network in the system
  - A sensor network is a group of (usually homogeneous) sensors deployed at multiple locations providing data streams that can be aggregated to obtain a set of combined sensor readings.
  - Creating a sensor network involves two main steps. First registering the sensor network details that are listed in Table 2. The Sensor Network references the sensor environment through the *Sensor Network Id*. A Wiki page is automatically created for every sensor network that gets created.

Stage 5: Registering sensors to a sensor network
  - Firstly the user has to create the set of sensors individually by repeating the steps (1 to 6) of case study 1 specifying the sensor network id. This links the sensors with the sensor network. The relevant sensor network Wiki page is then be updated with this information.

Stage 6: Query sensor data in a sensor network
  - The following sample query aggregates a set of sensors that belong to the same sensor network.
*SELECT Average (Value)**FROM Environment, Datastream, DataPoint* *WHERE sensorNetwork* = *‘SN-1’* *AND streamType* = *‘NO2’* *WIKI_WINDOW 1h*

### 5.2. Case Study 2: The Aggregation of Multiple Data Streams

Stage 1: View sensor data streams
  - When the users log in to WikiSensing they are able to view a list of sensors or sensor networks that were created by themselves as well as all the public sensors and sensor networks.
  - When the user, for example, requires obtaining the average temperature reading of South Kensington, London the relevant sensor data streams are aggregated to produce the output. Importantly, the system checks if the data streams are compatible for aggregation. If compatible they must then be checked for other disparities as data streams produced by different sensor devices may have different characteristics, for instance different output frequencies or different units of measurements.

Stage 2: Convert to a single unit of measurement
  - Firstly, if the units of measurements are different, WikiSensing automatically converts the values of the data streams to the unit of measure that is used by the majority of the data streams. If there are the same numbers of data streams with different units the system would then use a default unit of measurements. These rules are suppressed if the user specifies a unit of measurements in the query using the *WIKI_UOM* construct.

Stage 3: Sample different frequencies of data streams
  - There are two policies to handle disparity of frequency among data streams. The first policy samples the time frames of the data stream to fit the stream with the largest time interval. Table 3 illustrates this by combining the first streams readings at 10:27:30 and 10:28:0 to a single time frame of 10:28:0 so that it can be accurately mapped with the frequencies of the second data streams. This policy is applied when the user explicitly specifies the *WIKI_SAMPLE_STREAM* construct in the query.
  - The second, or default, policy where the user does not specify any construct in the query individually averages the data streams disregarding the differences of the frequencies.

Stage 4: Aggregate Queries
  - The following query outputs the average temperature reading at South Kensington, London. The *WIKI_PROPORTION* construct is used to indicate that the aggregated values must be based on the weighted mean of the specified attributes. The *WIKI_LOCATION* construct select records with in a location specified or the geographical coordinates. This query can be further extended using the *WIKI_RADIUS* construct that selects records within a radius (specified in meters) to the specified location or coordinates. The *WIKI_SAMPLE_STREAM* construct samples the data streams to match the stream with the largest frequency (Table 3).
*SELECT Average (Value)**FROM Environment, Datastream, DataPoint* *WHERE sensorType* = “*Temperature*’ *WIKI_LOCATION (Coordinates OR ‘South Kensington London’)* *WIKI_RADIUS 10* *WIKI_WINDOW 1 h* *WIKI_UOM Celsius* *WIKI_PROPORTION ON (DISTANCE, TIME)* *WIKI_SAMPLE_STREAM*
  - The user has the option to specify this query as continuous query with the construct *WIKI_CONTINUE_FOR* <*time interval in hours*>. This enforces the query to continuously run for the specified time period.
  - The queries explained so far are fetched data from two sources namely the meta-data of the sensor and their geographic information from a relational data base and the sensor reading from relational free data base.

### 5.3. Case Study 3: Creating a Virtual Sensor

Virtual sensors can be created when there is no physical sensor deployed at a specific location. This is useful when users require the aggregation of several data streams to be persistent.

**Figure 10.**
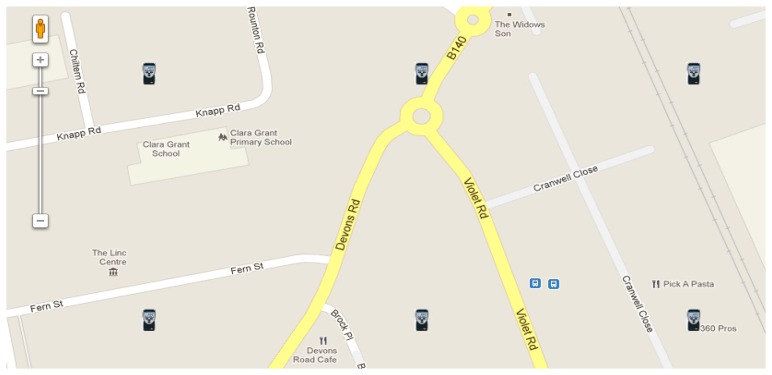
The WikiSensing map illustrating the deployment of sensors.

Stage 1: The search phase
  - The users can either view the WikiSensing map or query to check the locations of the physically deployed sensors. Figure 10 illustrates an instance of the WikiSensing map followed by an example query that would select certain sensors in a specific location.
  *SELECT EnvId, StreamId**FROM Environment, Datastream* *WHERE sensorType* = *‘SO2’* *WIKI_LOCATION* = *Coordinates OR Location_name*

Stage 2: Registering the details of a new virtual sensor
  - If the user requires a sensor reading from a particular location where a sensor is not physically deployed the user can create a virtual sensor in the system specifying its geographical details similar to registering a physical sensor described in use case 1 with the exception that the domain field must be set as ‘Virtual’. In addition users can specify the Virtual Sensor Reading to be either persistent or dynamic.
  - There are two categories of virtual sensors, the persistent virtual sensors which store the aggregated readings and the virtual sensors that generate readings dynamically. The readings of persistent virtual sensors can be traced for the origins of the contributing sensor data streams. For example, in the case where a doubt exists about a virtual sensor reading, this can be audited as the readings are recorded. In contrast dynamic virtual sensors produce their reading on request, and their output is generated by aggregating the data streams in real time.

**Figure 11.**
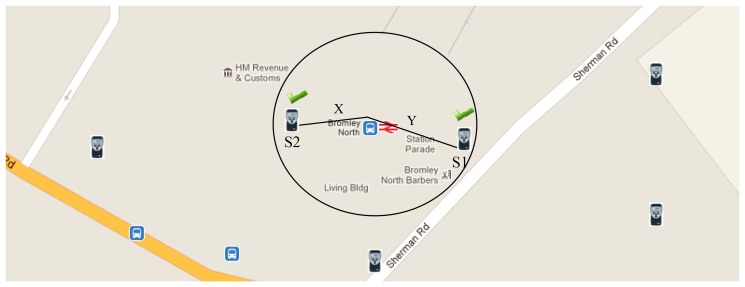
Selecting sensors to create a virtual sensor.

**Figure 12.**
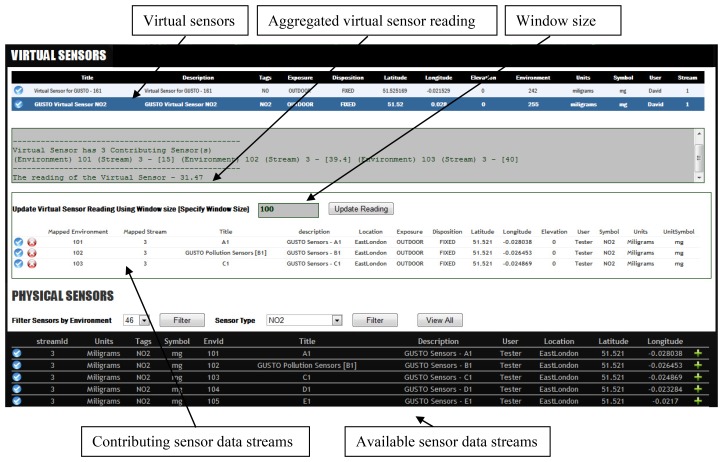
WikiSensing Interface for selecting sensor streams to create a virtual sensor.

Stage 3: Select and record the contributing sensors
  - The user can select nearby sensors that contribute to the newly created virtual sensor (Figure 11). The user has the option to add more sensors or remove any existing contributing sensors from the virtual sensor.
  - The sensors that contribute to a virtual sensor are recorded in a virtual sensor map table, whose fields are listed in Table 4. The optimize column is updated when the user explicitly requests the selected contributing sensors list to be optimized. The system updates this column with persistent virtual sensor identities that are already created using a subset of the selected sensors. The aim is to reduce the database reads using existing virtual sensor data streams that are already formulated.
  - Figure 12 illustrates the WikiSensing interface that enables users to add sensor data streams to a virtual sensor.

**Figure 13.**
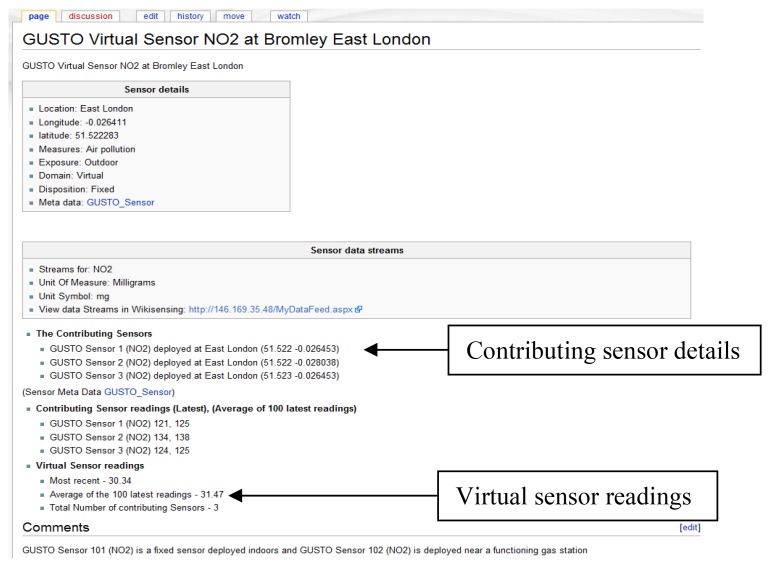
Wiki pages to record the creation of virtual sensors.

Stage 4: Aggregating the data streams of the contributing sensors
  - The system provides an aggregated sensor reading (of the contributing sensors) as the reading for the virtual sensor. The following query is an example that aggregates readings for a virtual sensor.
*SELECT Average (Value)**FROM Environment, Datastream, DataPoint* *WHERE EnvironmentIdentity IN {The set of contributing sensor environments}* *WHERE sensorType* = *‘Temperature’* *WIKI_RADIUS 10* *WIKI_WINDOW 1* *WIKI_UOM Celsius* *WIKI_PROPORTION ON (DISTANCE, TIME)* *WIKI_SAMPLE_STREAM*
  - In accordance to Figure 11 steams S1 and S2 are selected to contribute towards the virtual sensor. If the construct *WIKI_PROPORTION ON* is set to the distance and time the data streams are averaged based on the weighted mean on each of these attributes. This is calculated using the following formula:
    $$\bar{X}w=\frac{\sum \text{wx}}{\sum w}$$
    where *X̄w* is the weighted arithmetic mean, *x* stands for values of the items and *w* is the weight of the item.
  - The aggregation query that is responsible for obtaining virtual sensor readings is stored in the virtual sensor query table (Table 5). Users are able to update and save (validated before saving) these queries.
  - When the user completes the registration of a virtual sensor a Wiki page is automatically created and the information recorded (Figure 13). The Wiki page gets automatically updated when a user modifies the composition of the virtual sensor.

### 5.4. Case Study 4: Assessing the Trustworthiness of the Sensor information

- WikiSensing is accessible for any online use and in most cases the sensor data would need to be assessed for trustworthiness. The information that needs to be assessed is the sensor data streams and the annotations provided by the collaborating users. The trustworthiness of this information can be used in managing conflicts between different sources.
- There are various public sensor data streams available in the system. The users can view these data streams and their annotations with the purpose of understanding the information or with the intention of using it in their own analysis.

Stage 1: Identifying the information
  - When a user concentrates on a specific location to obtain a temperature sensor reading, identifies that there are two sensors deployed at that same location measuring the same attribute. The two sensors are providing conflicting readings of the temperature described in the following example.
*Temperature sensor 1 deployed at south Kensington station: 29 Celsius**Temperature sensor 2 deployed at south Kensington station: 21 Celsius*
  - The user also discovers that there are two annotations provided by separate users on a data stream that contradict each other.
*User 1: time stamp: 11/4/2012, the sensor ID 25 does not produce an accurate outcome of the temperature as it is located near a functioning refrigerator*.*User 2: time stamp: 10/4/2012, the sensor ID 25 is the primary sensor to obtain temperature readings for the William Penney building at Imperial College London*.

Stage 2: Calculate credibility of information
  - The user now has to make a decision in selecting a specific data stream of the two sensors as well as to know which annotation is valid. Hence the user can select the functionality for checking the credibility of sensors from the system.
  - When the check credibility functionality is executed the following ratings are automatically calculated.
    - Use the meta-data of the sensors to cross reference the capabilities of the sensors. In this case the sensor meta-information such as the sensitivity and the accuracy data are used to decide on which sensor is superior. This information is used to calculate the s*ensor reliability rating* that can be used to compare different sensors.
    - Aggregated readings of nearby sensors are obtained using the sensor readings of nearby sensors. This is compared with the readings of the sensors that need to be assessed for trustworthiness or to resolve a conflict. The output is the *distance of the sensor readings*.
    - To resolve conflicts between annotations provided by different users the system takes in to account the previous updates committed by those users as well as the background information such as qualifications and experience and calculate a *user reliability rating*.

Stage 3: The comparison
  - The s*ensor reliability rating*, *distance of the sensor readings* and the *user reliability rating* are used to create a single rating known as the *Credibility Rating.* This value is calculated by averaging the values obtained by the previous stage (Table 6). Users can compare these ratings to assess the trustworthiness of the information. In the case of managing conflicts these rating are compared with the sources that conflict each other.

Stage 4: The policy
  - Data streams, users and user annotations that are assessed through this process are annotated with this rating to help future users obtain a better understanding of the trustworthiness of this information.
  - The policy is that the credibility rating is a reflection on the trustworthiness of the information and therefore can be used to manage conflicts.

Stage 5: Log information in Wiki
  - This information is recorded in Wiki pages in order to obtain a trace or log of the type of method followed and the information that was used to assess the trustworthiness and to manage conflicts.

## 6. Experimental Evaluation

The experimental evaluation is designed to understand the attributes that affect the performance of the virtual sensors. The evaluation is based on different strategies that can be followed for aggregation queries and the storage for virtual sensor readings. The goal is to have an efficient methodology leading towards quicker responses to end users.

### 6.1. Improving the Performance of Aggregate Queries

We present two scenarios to demonstrate the methodology used by WikiSensing to improve the performance of aggregate queries. The performance is based on the response time of the queries and the improvement of the response time is a reflection of the decrease of the number of data base reads. The aim here is to identify strategies that reduce the number of database reads. A virtual sensor is an aggregation of one or more sensor data streams. The aggregate function takes a set of data streams and produces a single value that summarizes the information contained in the selected data streams [27]. In the case of virtual sensors that are persistent, it can record the results of the aggregation in the database.

#### 6.1.1. Scenario 1: Aggregate Sensor Data Streams to Create Virtual Sensors that Fully Overlap with Other Virtual Sensors

**Figure 14.**
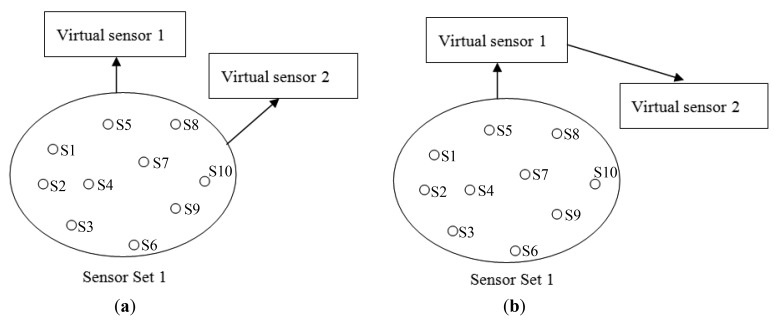
Aggregate sensor data streams to create virtual sensors that fully overlap with other virtual sensors (**a**) Using naïve methodology. (**b**) Using WikiSensing methodology.

Consider a scenario where a virtual sensor is already created using a set of sensors (Virtual sensor 1 in Figure 14(a)). A naïve strategy and the WikiSensing strategy are analyzed when the requirement for a second virtual sensor (Virtual sensor 2) arises. Firstly a naïve strategy creates the new virtual sensor by including all the required contributing data streams in the aggregate query (Figure 14(a)). This would not consider the fact that the fully overlapping virtual sensor 1 is a complete subset of virtual sensor 2. In contrast WikiSensing takes this fact in to account and create virtual sensor 2 by using the information in virtual sensor 1 (Figure 14(b)).

As the information of Virtual sensor 1 is persistent and cached the time involved in obtaining the result is expected to be less than a single database read. The aim of this strategy is to use existing persistent virtual sensors that are subsets of the newly created virtual sensor in order to reduce the number of data base reads. The trade-off using this strategy is the extra cost of storing the sensor readings. Hence it is important to identify the situations where persistent storage is suitable.

#### 6.1.2. Scenario 2: Aggregate Sensor Data Streams to Create Virtual Sensors that do not Fully with Overlap Other Virtual Sensors

**Figure 15.**
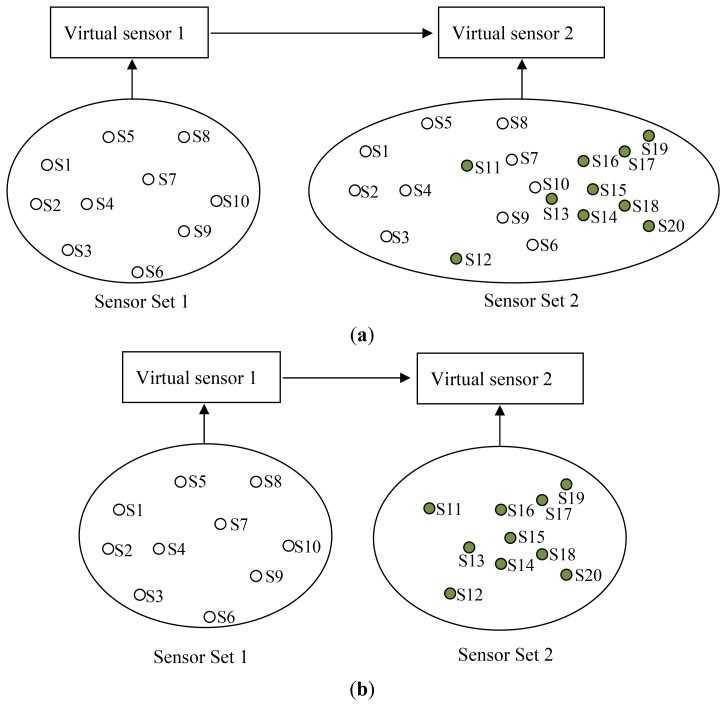
Aggregate sensor data streams to create virtual sensors that do not fully overlap with other virtual sensors (**a**) Using naïve methodology. (**b**) Using WikiSensing methodology.

Figure 15 depicts the requirement of a new sensor when the contributing streams do not fully overlap an existing virtual sensor (Virtual sensor 1). While a naive strategy would create new virtual sensor with all contributing sensors from scratch, WikiSensing uses the existing virtual sensor 1 and combine it with the other exclusive sensor streams. Similar to the first scenario, the readings of virtual sensor 1 can be taken from the cache and the rest of the reading can be fetched form the database.

### 6.2. Experimental Setup and Benchmark

The version of the WikiSensing system that is used for the experiment is implemented as a complete working system hosted on an IIS server running on a Windows server 2008 virtual machine in the IC-Cloud platform [28]. Test emulator that implements the Siege benchmark [29] is used to send requests and runs in another Linux Centos 5.4 virtual machine in the IC-Cloud. Siege is a regression testing and benchmarking utility that measures the performance of web applications and services.

The workload of the application tested obtains readings from physical sensors and virtual sensors that were created from a set of sensor data streams. The test emulator is run for a specific period of time and continuously generates a sequence of interactions that are initiated by multiple active sessions. After an interaction is completed, the emulator waits for a random interval before initiating the next interaction to simulate user's thinking time. Each experimental trial session is carried out for 300 seconds and three separate experiments are carried out. We are testing the performance by obtaining random readings from sensor data streams.

The first experiment measures the response times of a physical sensor by increasing the number of users accessing it. We use window sizes of 10 and 1,000 for a maximum of 1,000 simulated users.

The second experiment involves a single client accessing virtual sensor readings. This is further divided into 2 trials where we test with a window sizes 10 and 1,000 sensor readings. Each trial is tested with different workloads that are the naïve approach and the WikiSensing strategies based on a 100%, 80%, 50% and 20% overlap of sensors.

The third experiment has the same parameters as the previous one, except the fact that it is tested using multiple simulated users with active sessions. The first trial simulates 100 clients concurrently accessing the system with the gradual increase of the contributing sensors. The second trial gradually increases the number of clients that access a virtual sensor created with 50 sensor data streams.

The test emulator based on the Siege benchmark outputs the response time for each experimental scenario. The emulator makes an HTTP request for a web page that invokes a web service function. The response time is calculated from the start of the invocation till the function returns a value and is loaded into the web page. The time for each execution is summed and averaged to obtain uniform reading.

#### 6.2.1. Experiment 1: Measure Response Time of a Physical Sensors Accessed by an Increasing Number of Clients

We test the response time of obtaining readings form a physical sensors with the increase of the number of users. This results in increasing the number of concurrent users that access a single sensor stream with a window size of 10 and 1,000.

**Figure 16.**
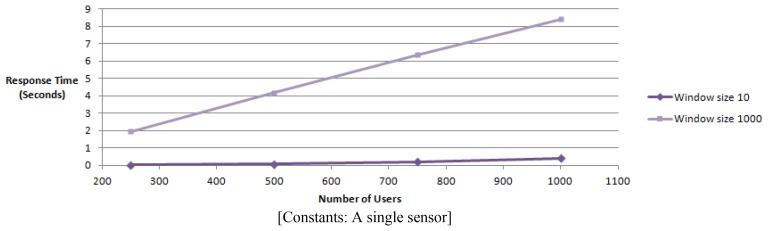
Response times for querying a single physical sensor by increasing the number of clients.

The number of concurrent clients are increased from 250 to 1,000. The response time *R(t)* has a dependency on the number of concurrent users *(X)* and the window size *(Y)*, *R(t)* = *f(X,Y)* according to the graph (Figure 16).

#### 6.2.2. Experiment 2. Measuring Response Time of Virtual Sensors Accessed by a Single Client with Respect to the Increase of the Contributing Sensor Data Streams

We measure the response time for obtaining an aggregate reading of a virtual sensor with respect to the increase of the number of contributing sensors. The aggregate reading is a combined or averaged single value of the contributing sequential data streams. It tests a single client accessing the virtual sensors reading by gradually increasing the number of contributing sensors from 10 to 140. The different workloads are the naïve approach where all records are fetched from the database, 100% overlapping where the information is picked form the server cache and 80%, 50% and 20% overlapping where the data is fetched directly from the database.

Virtual sensor readings are cached when the user makes a request for that sensor. If the data is not cached it is then fetched from the database. Overlapping is dealt with in WikiSensing as illustrated in Figure 15(b). For example, if the overlapping is 80% for a virtual sensor it obtains the overlapped portion using a single database read (or directly from the cache if the information is cached) and gets the rest (20%) of the reading from the other data streams.

**Figure 17.**
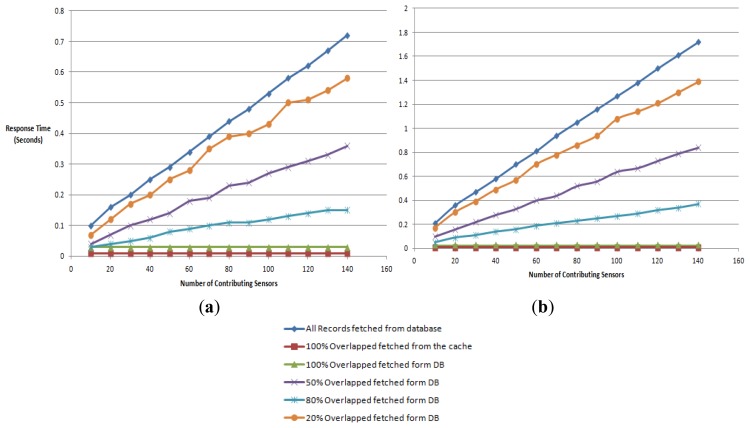
Comparing the response times for querying a single virtual sensor (**a**) With a window size of 10. (**b**) With a window size of 1,000.

We have used 2 Trials with windows sizes 10 (Figure 17(a)) and 1,000 (Figure 17(b)). The aim of changing the window size is to alternate the amount of sensors reading that are selected for an aggregate query. For instance, a window size of 10 selects the 10 most up-to-date sensor readings for the aggregate query.

The response times for both the scenarios with a 100% overlap (fetched from the database and the cache) were constant throughout the experiment and returned response times of 30 and 10 milliseconds. With a window size 10, the response time of a single virtual sensor is in the range of 60 to 20 milliseconds for the naïve, 80%, 50% and 20% overlapping workloads. The performance for a single virtual sensor when used with window size of 1,000 is in the time span of 110 to 30 milliseconds for the respective workloads.

The response time for the virtual sensors readings *R(t)* has a dependency on the number of contributing sensors *(X)* and the window size *(Y)*, *R(t)* = *f(X,Y).* When comparing the results of the two window sizes the different strategies have responded in similar fashion. The main difference here is the response time increases when using a window size of 1,000. The response time of the 50% overlapped workload at 140 sensors (window size 10) is 370 milliseconds. This response time increases when the overlapping is reduced and increases when the overlapping is reduced. This is due to the impact of the increase in the number of database reads. Thus the decrease of overlapped sensors constitutes a 60% change of the response time. The same situation prevails with a window size of 1,000 as well.

#### 6.2.3. Experiment 3: Measuring Response time of Virtual Sensors Accessed by 100 Concurrent Clients with Respect to the Increase of the Contributing Sensor Data Streams

**Figure 18.**
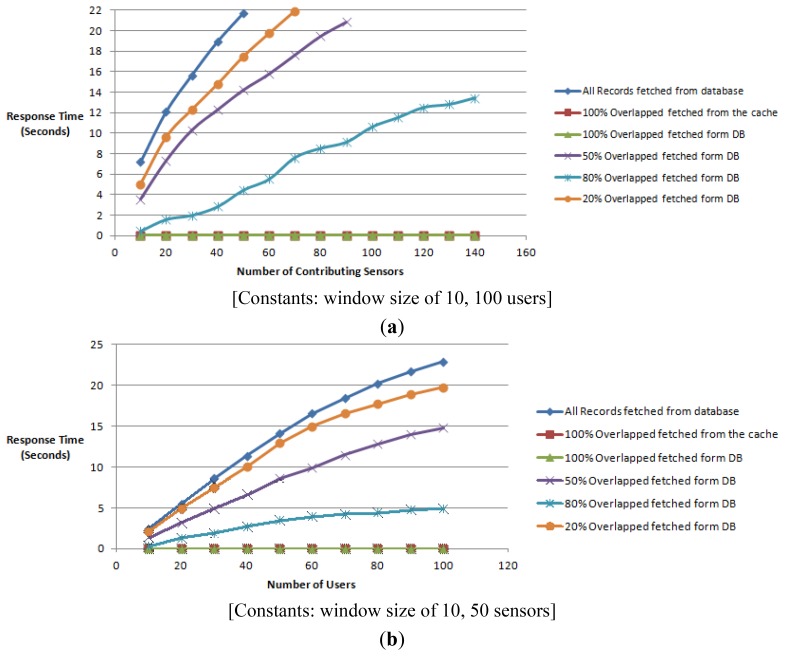
Response times for querying a single virtual sensor (**a**) Increasing the number of contributing sensors with 100 concurrent users (**b**) Increasing the number of users with 50 sensors.

This test simulates a case where a popular virtual sensor is accessed by many users. In the first trial we measure the response time of an aggregate reading of a virtual sensor with 100 clients accessing the same set of data concurrently. The second trial records the response time by increasing the number of clients from 10 to 50 and keeping the number of contributing sensor data streams constant at 50. In both trials we use a window size of 10. This experiment mainly focuses on testing the response and the scalability of the system. The graph in Figure 18 depicts the bottlenecks with the scenarios when fetching data where the overlapping does not exceed 50%. The scenarios that 100% overlap fetched from the database and the memory cache returned the constant response times ranging from 30 and 10 milliseconds throughout this experiment.

The test emulator times-out due to memory limitation when using a traditional naïve strategy when the number of sensors exceeds 50 as depicted by the graph in Figure 18(a). Clearly the strategy followed by WikiSensing to use the principles of overlapping scales better that the traditional approach as the response times are comparatively less.

The response time for the virtual sensors readings *R(t)* has a dependency on both the number of contributing sensors *(X)* the window size *(Y)* and the number of concurrent users *(Z)*, *R(t)* = *f(X,Y,Z)*. As the data access intensifies with 100 concurrent users the response time tends to increase and the performance is diminished in the strategies where there is 50% or less overlapping. Form these experiments we can conclude that, response time for virtual sensor readings:
*R(t)* (Naïve) = *N* * *d(t)* + *a(t)**R(t)* (WikiSensing, when cached) = *c(t)* + *(N - O) d(t)* + *a(t)**R(t)* (WikiSensing, when fetched form database) = *d(t)* + *(N - O) d(t)* + *a(t)*where *N* is the required number of sensors, *O* is the overlapped number of sensors, *d(t)* is the time to fetch record from database, *c(t)* is the time to fetch record from cache, *a(t)* is the time to process the aggregation.

The other factors that affect the response time of such an HTTP request are the performance of the browser, the speed of the Internet connection, the local network traffic, the load on the remote host, and the structure and format of the web page requested [30]. Taking the time cost of all these factors as *X*, the total response time is = *R (t)* + *X*.

## 7. Summary and Discussion

The WikiSensing system at present can be used with sensors that produce data streams. At this stage the system does not support other formats such as images, text messages or audio or video files (security cameras, audio sensors). We realize the significance of widening the scope in order to store different types of media as a future enhancement of WikiSensing.

A major goal of sensor data management is that it can be used by different applications that provide users with useful information. For example, consider an application that reads the pollution data from a sensor data management system and provides warning to asthmatic users, or a heart beat ECG monitoring application that alerts its users when values reach certain thresholds. These are examples of applications that can use WikiSensing as a repository for their sensor data and make use of the API services it provides. The applications usually compare readings against a prescribed threshold value. We aim to support more complicated applications that depend on the readings of multiple sensors and require several parameters for decision making. For instance, contemplate an application that outputs the disturbance noise levels of a room at a particular location. To provide an accurate reading this application requires the information on the noise levels of the traffic, distance to bus stops, the type of traffic that passes through (as in heavy or light vehicles), thickness of double glazing of windows, the elevation of the room, the reliability of the sensors and so on. WikiSensing currently stores sensor geographical data variety of sensor meta-information but its goal is to store more domain specific information in order to support complicated applications as the one previously mention.

When considering the openness of WikiSensing for online collaboration it facilitates creating virtual sensors, updating information as well as allowing users to provide their feedback or comment on the existing sensor data. A current limitation of the system is based on the granularity of commenting on this data. The feedback functionality of WikiSensing enables users to add comments to a particular data stream but cannot add annotations to a specific point of a graph that represents a sensor reading. This is useful to understand more about sensor data streams and can be a part of the functionality, incorporated in the future.

Calibration details are deemed important in understanding the quality of sensor measurements. We plan to incorporate calibration details as a separate entity in the data model linked to the sensor data points as it relates to periods of measurement of a sensor. This information can be a part of the sensor meta-data that can be used as a metric in assessing the trustworthiness of sensors.

The system currently supports a single trustworthiness score. However this score can be categorized in to several aspects of a sensor, for example such as the reliability, accuracy or calibration. The system can provide an ontology containing various attributes and its relationships with each other. For instance, the reliability of a sensor could not be the same when it is located outdoors as opposed to being placed indoors. Hence the ontology could contain definitions for both these scenarios as the same attribute may have different values depending on the circumstance. The work described by [31] is with particular interest as it presents information on developing ontologies for heterogeneous sensors. We can also use the JCGM VIM [32] standard terminology for selecting sensor attributes in designing the ontology.

The functions currently supported by the system are based on simple aggregations such as summing and averaging. There is a limitation imposed on enabling users to define their own functions and being able to execute them at the centralized server, as it may poses issues on performance and security. In order to evade there problems the system API allows users to obtain the data streams and perform complex functions locally. The research work by [33] can be considered when enhancing the system to support complex aggregation functions.

## 8. Conclusions and Future Work

### 8.1. Conclusions

This paper has introduced a new collaborative approach for sensor data management known as WikiSensing. It has presented an architectural design and described the implementation details for a collaborative sensor data management system. The advantage of WikiSensing is based on incorporating online collaboration into sensor data management. Online collaboration is used in WikiSensing to annotate, update and share sensor information as well as in creating virtual sensors. The virtual sensor concept is an extremely useful feature that provides sensor readings using existing sensor data streams. The main challenges in sensor data management and online collaboration is due to the large amounts of sensor data and the inability to demonstrate the trustworthiness of the shared information. This research has addressed some of these challenges towards developing a successful collaborative sensor data management system.

We anticipate that the convergence of online collaborations with sensor data management can enable better use and understanding of the vast amounts of sensor information. Further the efforts required are considerably lower due to the collaborative nature and the involvement of users with experience and knowledge on sensors and their deployments.

### 8.2. Future Work

We plan to concentrate on enhancing the response time of the extensively used aggregate queries as well as implementing a mechanism to trace the developments of the virtual sensors. From an analytical perspective we are working on building a wiki analytical layer for the sensor and wiki data that can markup the information using a universal methodology. In the short term out future work will focus on the following aspects.

An important future development would be to trace the modifications of virtual sensors. Hence we plan to extend the data model in order to maintain a record of changes applied to virtual sensors. A potential source for this information could be the updates applied to the virtual sensor network and the virtual sensors query entities. The work done by [34] highlights the challenges in managing historical sensor information and can be used as the basis for this development.

We hope to reduce the response time of aggregate operations by using the MapReduce MongoDB [35] for batch processing of data. This is similar to Apache Hadoop (hadoop.apache.org) but uses distributed processing of large data sets across clusters of computers. MapReduce in MongoDB processes the input from a collection and outputs it to a collection. This can be used for the aggregation queries especially when they involve combining a large number of data streams. This relates to the work of [36] that proposes a scalable platform for network log analysis, which targets for fast aggregation and agile querying.

Our main objective is to use the gathered sensor data and put it into further analysis with the goal of helping users to obtain useful insights. For example, it could be useful to know whether there is a relationship between the temperature of the environment and the pollution levels of NO or ozone or between the noise levels and the prevailing traffic. The data in the system must therefore be transformed into a suitable format in order to make further use of it and the system must provide the suitable functionality. This can be supported by adding a new layer to out architecture.

**Figure 19.**
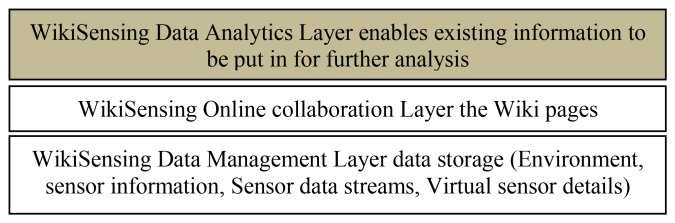
The proposed new layer for organizing the sensor data and user annotations.

The proposed new layer (highlighted in Figure 19) would enable the existing data and information to be formatted and annotated based on a standard markup. The functionality of this tier would be able to extract and use the information from the Wiki pages created as result of online collaborations. The goal is to annotate this information so that is can be further analyzed thereby increasing the chances of obtaining useful insights form this rich set of underlining sensor data.

## Figures and Tables

**Table 1.** The list of fields involved in registering sensors in WikiSensing.

**Field**	**Mandatory**	**Domain**	**Description**

Environment Identity	Yes	Number	The identity of the environment
Environment Name	Yes	String	The name of the environment
Feed Description	No	String	A description of the data streams and its measurements
Location Description	Yes	String	Details of the deployed location of the sensor
Access Right	No	Boolean	Public or private, and private by default
Latitude	No	Float	Latitude of the sensor environment
Longitude	No	Float	Longitude of the sensor environment
Elevation	No	Float	Elevation of the sensor
Exposure	No	String	Whether the sensor location is indoor or outdoor
Disposition	No	String	Whether the sensor location is fixed or mobile
Domain	No	String	Whether the sensor is physical or virtual
Virtual Sensor Reading	No	String	Whether the virtual sensor readings are stored
Sensor Network	No	String	The network Identity of the sensor
Data Stream Identity	Yes	String	The identity of the data stream
Stream Type	Yes	String	The type of attribute that is measured
Unit of Measure	Yes	String	The measuring unit of the data stream

**Table 2.** The list of fields to register a sensors network.

**Field**	**Mandatory**	**Domain**	**Description**

**Sensor Network Id**	Yes	Number	The identity of the sensor network
**Sensor Network Name**	Yes	Number	The name of the sensor network
**Description**	Yes	String	A description about the sensor network
**Purpose**	No	String	The motivation for creating a sensor network

**Table 3.** The sampling of the frequency of multiple data streams.

**Frequency of submitting readings every 30 seconds**	10:27:30	10:28:0	10:28:30	10:29:0	10:29:30	10:30:0	10:30:30	10:31:0
**Frequency of submitting readings every 60 seconds**		10:28:0		10:29:0		10:30:0		10:31:0
**Sampled frequency of aggregated stream**		10:28:0		10:29:0		10:30:0		10:31:0

**Table 4.** The list of fields to register a virtual sensors network.

**Field**	**Mandatory**	**Domain**	**Description**

Virtual Sensor Environment Identity	Yes	Number	The identity of virtual sensor environment
Contributing Sensor Environment Identity	Yes	Number	The identity of contributing sensor environment
Datastream identity Virtual Sensor	Yes	Number	The identity of the data stream of virtual sensor
Datastream identity Contributing Sensor	Yes	Number	The identity of the data stream of contributing sensor
Optimize	No	Number	Set of identities of selected virtual sensors that is used to optimize performance.

**Table 5.** The list of fields in the virtual sensor query table.

**Field**	**Mandatory**	**Domain**	**Description**

Virtual Sensor Environment Identity	Yes	Number	The identity of the virtual sensor
Datastream identity Virtual Sensor	Yes	Number	The identity of the data stream of virtual sensor
Query	Yes	String	The SQL of the aggregate query

**Table 6.** Calculate the credibility rating.

**Source**	**Sensor reliability rating (out of 1)**	**Distance of sensor reading (out of 1)**	**User reliability rating (out of 1)**	**Credibility rating (out of 1)**
**A conflict between two data streams**				
Temperature sensor 1	0.1	0.2	NA	0.15
Temperature sensor 2	0.7	0.9	NA	0.8
**A conflict between annotations from two users**				
User 1 on Sensor 25	0.5	0.6	0.5	0.54
User 2 on Sensor 25	0.5	0.6	0.2	0.26
